# “Dirty Dancing” of Calcium and Autophagy in Alzheimer’s Disease

**DOI:** 10.3390/life13051187

**Published:** 2023-05-15

**Authors:** Hua Zhang, Ilya Bezprozvanny

**Affiliations:** 1 Department of Physiology, UT Southwestern Medical Center, Dallas, TX 75390, USA; hua.zhang@utsouthwestern.edu; 2 Laboratory of Molecular Neurodegeneration, Peter the Great St. Petersburg State Polytechnical University, St. Petersburg 195251, Russia

**Keywords:** ryanodine receptor, autophagy, calcium signaling, calcineurin, Alzheimer’s disease

## Abstract

Alzheimer’s disease (AD) is the most common cause of dementia. There is a growing body of evidence that dysregulation in neuronal calcium (Ca^2+^) signaling plays a major role in the initiation of AD pathogenesis. In particular, it is well established that Ryanodine receptor (RyanR) expression levels are increased in AD neurons and Ca^2+^ release via RyanRs is augmented in AD neurons. Autophagy is important for removing unnecessary or dysfunctional components and long-lived protein aggregates, and autophagy impairment in AD neurons has been extensively reported. In this review we discuss recent results that suggest a causal link between intracellular Ca^2+^ signaling and lysosomal/autophagic dysregulation. These new results offer novel mechanistic insight into AD pathogenesis and may potentially lead to identification of novel therapeutic targets for treating AD and possibly other neurodegenerative disorders.

## 1. Introduction

Alzheimer’s disease (AD) is an age-related brain disorder that causes progressive neurodegeneration predominantly in the cortical and hippocampal brain regions. Major hallmarks of AD are the progressive impairment of memory storage and accumulation of fibrillary amyloid plaques in patient’s brains. Despite decades of research and effort, there is still no effective disease-modifying treatment for AD. Although amyloid pathology is a hallmark and defining feature of AD, targeting amyloid pathways has been very challenging due to low efficacy and serious side effects. Therefore, it is important to explore alternative approaches for treating memory loss in AD [1]. Increasing studies suggest that Ca^2+^ dysregulation in AD plays an important role in AD pathology and is associated with other AD abnormalities, such as excessive inflammation, increased ROS, impaired autophagy, neurodegeneration, and synapse and cognitive dysfunction [2,3,4,5]. Autophagy is important for removing unnecessary or dysfunctional components and long-lived protein aggregates, and a substantial amount of evidence in both AD patients and AD animal models indicates autophagy dysregulation plays an important role in AD pathogenesis [6,7,8,9,10,11,12,13,14,15]. Autophagy can also be regulated by intracellular Ca^2+^ signals arising from different organelles, including ER, mitochondria and lysosomes [16,17,18]. The majority of Ca^2+^ release from the ER is mediated by two families of Ca^2+^-permeable channels, inositol 1,4,5-trisphosphate receptors (InsP_3_Rs) and ryanodine receptors (RyanRs). The role of InsP_3_Rs in regulation of autophagy had been intensively studied in non-excitable cells, and InsP_3_R-mediated Ca^2+^ signals have been suggested to be involved in both inhibitory and stimulatory effects on autophagy [17,19,20,21,22,23]. Recently, the role of RyanR in regulating lysosomal acidification [24] and autophagy [25,26,27] is receiving greater attention. On another hand, some recent studies suggested that changes in lysosomal acidification may affect intracellular Ca^2+^ signaling by modulating activity of TRPML1 lysosomal Ca^2+^ channels [28,29]. In this review, we will focus on emerging interplay between dysregulated Ca^2+^ signaling and dysfunction of the lysosomal/autophagic system in AD neurons.

## 2. Intracellular Calcium Signaling Dysregulation in AD

There are two types of intracellular Ca^2+^ release channels in neurons—InsP_3_Rs and RyanRs. There are three isoforms of InsP_3_Rs, the predominant one in neurons being InsP_3_R type 1. It is known that expression of FAD PS1 mutants in *Xenopus* oocytes potentiates InsP_3_-mediated Ca^2+^ release [30], and that InsP_3_R activity can be potentiated in PS1(M146V) knock-in mice [31]. Similar potentiation was also reported in human lymphoblasts expressing FAD mutant PS1-M146L [32]. The InsP_3_Rs are known to be enriched in MAMs, and up-regulated Ca^2+^ release from ER through InsP_3_R can overload mitochondria, cause openings of the mitochondrial permeability transition pore (PTP), cause a reduction in the inner mitochondrial membrane potential, result in drop in NADH and ATP levels, and potentially lead to cell death [33]. Computational modeling of single InsP_3_R1 channel activity showed that significantly lower InsP_3_ levels are needed to induce the same level of InsP_3_R1 channel activity in the presence of FAD-PS1 mutants [34]. Besides FAD-PS1, Aβ can also affect InsP_3_R function, and it was reported that Aβ42 can induce a cytosolic Ca^2+^ increase in both an InsP_3_R-dependent and InsP_3_R-independent manner [35]. Genetic reduction InsP_3_R1 by 50% normalized exaggerated Ca^2+^ signaling in PS1M146V knock-in mice and restored hippocampal long-term potentiation (LTP). In 3xTg mice, reduced InsP_3_R1 expression reduced amyloid β accumulation and tau hyperphosphorylation and restored hippocampal LTP and memory deficits [36]. Proper InsP_3_Rs function is important to maintain spine morphology, synaptic plasticity and memory consolidation [37,38], and limited inhibition of InsP_3_R may be considered as a potential therapeutic approach for AD.

InsP_3_R1 plays an important role in Ca^2+^ signaling in cerebellar Purkinje cells, but in most other neurons RyanRs play a predominant role in control of intracellular Ca^2+^ levels. There are three structurally similar mammalian isoforms of RynaRs—RyanR1, RyanR2 and RyanR3. RyanR1 was initially found in skeletal muscles but also expressed in cerebellar Purkinje cells. RyanR2 is expressed in cardiac muscle cells and in most neurons. RyanR3 is also expressed in neurons, but RyanR2 is the most dominant neuronal isoform with the exception of cerebellar Purkinje cells [39,40,41,42]. Neuronal RyanRs are activated by Ca^2+^-induced Ca^2+^ release (CICR) mechanisms in response to initial Ca^2+^ influx via voltage-gated Ca^2+^ channels or NMDARs. RyanRs can also open in response to endoplasmic reticulum (ER) Ca^2+^ overloads in resting neurons [43]. These spontaneous Ca^2+^ release events (“Ca^2+^ sparks”) act to control ER Ca^2+^ levels and also contribute to setting basal levels of cytosolic Ca^2+^ in resting cells.

There is extensive evidence that activity and expression of RyanR2 is elevated in AD neurons. The expression and function of RyanR2 is increased in animal models with familial AD (FAD) and in early stages of sporadic AD in patients [42,44,45,46,47,48,49,50]. Aging is the most important risk factor for sporadic AD, and enhanced activity of RyanR may play a critical role in aging-related cognitive impairments [51]. Microarray analysis shows that RyanR2 expression continues to increase in the hippocampus from 6 months of age and onwards in mice, while the level of neuronal FKBP1b protein which binds to RyanR2 and stabilizes their opening is high at early developmental stages and begins to decrease at approximately 3 months of age [52]. This down-regulation continues throughout the aging process, leading to minimal amounts of FKBP1b being detected at 23 months of age, likely leading to further RyanR2 overactivation [52]. Consistent with the importance of RyanR overactivation for AD pathogenesis, pharmacological inhibitors of RyanR such as dantrolene demonstrated beneficial effects in a variety of AD cellular and animal models [45,48,53,54]. In our previous studies we used a genetic strategy and evaluated effects of RyanR3 knockout in AD mouse models, and we found a dual role for RyanR3 in AD pathology depending on the different stages of the disease [42]. Beneficial effects of a gating mutation of RyanR2 in AD mouse models have been previously demonstrated [55,56,57]. However, the mechanisms linking RyanR overactivation with AD pathology are less clear. Some investigators suggested a mechanism that involves the restoration of neuronal hyperexcitability in AD mice [42,55,56,57], some suggest that blocking RyanR-mediated Ca^2+^ leakage may lead to decreased endoplasmic reticulum (ER) stress [58], some focused on the role of RyanR-mediated Ca^2+^ changes in synaptic functions [54], and some suggested dantrolene can decrease β- and γ-secretase activities and APP phosphorylation by affecting Cdk5 and GSk3β kinase activities [45].

## 3. Dysregulation of Autophagy in AD

Autophagy is a process that maintains healthy cells, organelles, proteins, and nutrient homeostasis in living organisms. Three types of autophagy are observed in mammalian cells depending on the mode of substrate delivery: macroautophagy, chaperone-mediated autophagy, and microautophagy [59]. In macroautophagy, a double-membrane vesicle known as an autophagosome engulfs its targets by isolating a portion of the cytoplasm. The autophagosomal membrane fuses with lysosomes to form autophagic vesicles, the contents of which are degraded by lysosomal proteases. In microautophagy, substrate proteins are internalized into lysosome lumen via membrane invagination. In chaperone-mediated autophagy (CMA), substrate proteins are translocated across the lysosomal membrane by chaperone proteins [60]. In most cases, term “autophagy” refers to macroautophagy.

Autophagy is a conserved cellular process that removes damaged or nonfunctional cellular organelles to recycle the materials and maintain the cell function and efficiency. It also destroys pathogens that invade the cells in order to protect the cells, and plays an important role in removing long-lived proteins and aggregates that help to maintain cell homeostasis [61]. Neurons are post-mitotic and long-lived cells; misfolded proteins and damaged organelles cannot be diluted through cell division and thus they are more easily affected by protein homeostasis impairment. Since Atg5^−/−^ and Atg7^−/−^ mice die soon after birth, researchers made neural-cell-specific Atg5 or Atg7 knockout mice. Both mice show dramatic abnormal intracellular protein accumulation and form intraneuronal aggregates and inclusions that increased in size and number with aging. They also show progressive neurodegeneration and cell death, which suggests autophagy is essential for normal neuronal function and survival [62,63]. Neurons are highly polarized cells with axon and synaptic terminals far away from the cell body, which is the primary site for protein synthesis and degradation. Recently, researchers found autophagosomes are constitutively formed at synaptic sites in the distal axon, with new autophagosomes engulfing soluble and aggregated proteins as well as mitochondrial [64], ER [65] and damaged synaptic vesicles [66]. They rapidly fuse with a late endosome or lysosome, and subsequently retrograde transport along the axon to the cell body. This conserved mechanism plays an important role in the maintenance of synaptic homeostasis [67,68]. Specific deletion of Atg7 in Purkinje cells initially causes cell-autonomous progressive degeneration of the axon terminals with little sign of dendritic or spine atrophy, suggesting that axon terminals are much more vulnerable to autophagy impairment than dendrites [69]. Autophagy can regulate synaptic plasticity by modulating synaptic transmission through removal of SVs and their associated proteins [70] or through controlling the axonal ER [65]. interestingly, it was reported that autophagy protein LC3B is also an RNA-binding protein that can trigger rapid mRNA degradation to control local protein synthesis [71]. Even though the main sites of autophagosome biogenesis are at distal axons, autophagy in postsynaptic sites were also recently reported to play important role. It was shown that NMDAR-dependent LTD induction triggers a profound reorganization of PSD-95, which requires the autophagy mechanism to remove the T19-phosphorylated form of PSD-95 from synapses [72]. It was also shown that during NMDAR-LTD, key postsynaptic proteins are sequestered for autophagic degradation, pharmacological inhibition of AV biogenesis, or knockdown of atg5. Specifically, postsynaptic pyramidal neurons in the CA1 area abolish LTD. These data suggest that local autophagy of postsynaptic proteins in dendrites involve LTD expression [73]. Synaptic plasticity is the foundation of learning and memory, which is impaired in AD, so autophagy dysregulation will also play important role in AD [7].

Autophagy dysregulation plays an important role in AD pathogenesis according to the studies of both AD patients and animal models. Using immunogold staining with compartment-specific markers and electron microscopy of AD patients’ brain samples, it was demonstrated that autophagysome, multivesicular bodies, multilamellar bodies, and cathepsin-containing autophagosomes were accumulated in dystrophic neurites and synaptic terminals in AD brains [10]. APP/PS1 double transgenic mice also showed that autophagosomes and late autophagic vacuoles (AVs) accumulate markedly in dystrophic dendrites, implying an impaired maturation of AVs to lysosomes. In the hippocampus of young (4- to 6-month-old) PS1(M146L)/APP(751SL) mice models, many autophagic vesicles accumulated in the dystrophic neurites and presynaptic terminals surrounding amyloid plaques, and the autophagosome marker LC3 was also increased around plaques [13]. Interestingly, silver-enhanced immunogold labeling revealed that APP preferentially localized to the AVs within plaque-associated dystrophic neurites [13].

Autophagy is the main mechanism for regulating the processing of APP and intracellular Aβ peptide. It was discovered that AVs enriched Aβ, βCTF, and also the components of the γ-secretase complex, and that Aβ production increased after macroautophagy was acutely stimulated, implying that the cleavage of APP occurs in the AVs [12]. Interestingly, autophagosomes can also transport BACE1 and regulate BACE1 trafficking and degradation. Autophagic vacuole-associated BACE1 is accumulated in the distal axon of Alzheimer’s disease-related mutant human APP transgenic neurons and mouse brains which exacerbates the AD pathological changes [74]. Therefore, it is possible that accumulated β-secretase, γ-secretase complex and APP in autophagic vacuoles enhances the β and γ processing, resulting in Aβ overproduction. Autophagy also possibly participates in the secretion of Aβ [75]. When transgenic mice overexpressing an amyloid precursor protein (APP) were crossed with the mice lacking autophagy in excitatory forebrain neurons, the amyloid plaques were dramatically reduced, while intraneuronal Aβ accumulated in the perinuclear region and caused neuron degeneration and cognitive dysfunction [76]. Moreover, it was shown that these changes were due to reduced Aβ secretion, suggesting that autophagy is important for Aβ secretion [76].

Autophagy also plays an important role in the degradation of both soluble and insoluble Tau, another signaling component of AD pathology. Wang et al. demonstrated in an inducible neuronal cell model of tauopathy that the autophagy-lysosomal system contributes to both Tau fragmentation into pro-aggregating forms and to clearance of Tau aggregates. Inhibition of macroautophagy enhances Tau aggregation and cytotoxicity. Unlike the proteasome, autophagy degrades tau regardless of phosphorylation. Tau phosphorylated at the KXGS motifs of the repeat domain, which cannot be degraded by the UPS, is efficiently degraded by autophagy [77]. However, they also found that N terminus truncated species are preferentially trapped by CMA, and C terminal truncation occurred. The C terminal-truncated tau promoted tau aggregation. Even though soluble tau, aggregated tau, and C terminal-truncated tau mainly degraded through the autophagy lysosome system, full-length tau is preferentially digested through proteasomes [78]. Recently, it was reported that a large fraction of neuronal tau is degraded by CMA, whereas upon acetylation, tau is preferentially degraded by macroautophagy and endosomal microautophagy [79]. Consistent with these findings, trehalose and rapamycin have been shown to result in a significant reduction in cortical tau tangles, less tau hyperphosphorylation, and lowered levels of insoluble tau in the forebrain in P301S mutant tau transgenic mice by stimulation of autophagy [80,81]. Secreted soluble tau species spread trans-cellularly were reported in AD, and data have shown that autophagy inducers can promote tau secretion and knockdown. Beclin1 or autophagy inhibitors can inhibit tau secretion, and researchers have further identified that six isoforms of tau protein are secreted in an autophagy-dependent manner [82,83]. Accumulated data showed that secreted Tau contributes to synaptic impairment in AD, especially in GABAergic transmissions [84,85].

## 4. Mechanisms Underlying Autophagy Impairment in AD

Despite extensive evidence regarding dysregulation of autophagy in AD, there is no clear understanding of mechanisms that cause this impairment. The build-up of AVs in neurodegenerative diseases may reflect enhanced autophagy induction, impaired later lysosomal degradation steps in the autophagic pathway, or a lower rate of autophagy initiation combined with insufficient lysosome fusion and digestion [7]. Several reports suggest that autophagy induction is impaired in AD neurons. Beclin1, a protein with a key role in autophagy initiation, was decreased in affected brain regions of AD patients early in the disease process [86] and the expression of p62, an autophagic cargo receptor, was reported decreased in AD brains relative to age-matched controls [87]. There is a report that autophagy is transcriptionally down-regulated during normal aging in the human brain, and in contrast to normal aging, they observe transcriptional up-regulation of autophagy in the brains of AD patients, suggesting that there might be a compensatory regulation of autophagy [88]. A critical role of lysosomal proteolytic failure in AD neurons has been suggested by other investigators [89,90]. Presenilin1 (PS1) mutations are the most common cause of early-onset familial AD (FAD), in addition to its role as a catalytic subunit of the γ-secretase complex, PS1 is also essential for v-ATPase targeting to lysosomes, lysosome acidification, and proteolysis during autophagy. Fibroblasts from patients with FAD caused by PS1 mutations also exhibit markedly defective lysosome acidification and autolysosome maturation, a similar mechanism to that seen in PS1-null cells [91]. However, there are also reports that endo-lysosomal dysfunction in PSEN-deficient cells is due to lysosomal calcium homeostasis defects, not proton pump defects [92,93] (please see discussion in [94]). An APP-dependent compromise of lysosomal acidification was also reported in multiple AD mouse models in which FAD-mutant APP alone is expressed [95]. The mechanism for this lysosome failure is not clear, although it has been proposed that decreased expression of the motor proteins kinesin and dynein can induce axonal transport impairments and further impair lysosome trafficking, maturation and function [96]. Defects of lysosomal acidification observed in AD models suggest that stimulation of lysosomal acidification should elicit beneficial effects in AD. This can be achieved, for example, by stimulating the activity of lysosomal v-ATPase by disrupting its association with STK11IP, a recently identified lysosome-specific substrate of mTORC1 that regulates lysosomal acidification [97].

## 5. Dysregulated Ca^2+^ Signaling and Autophagy Defects in AD

**Figure 1:**
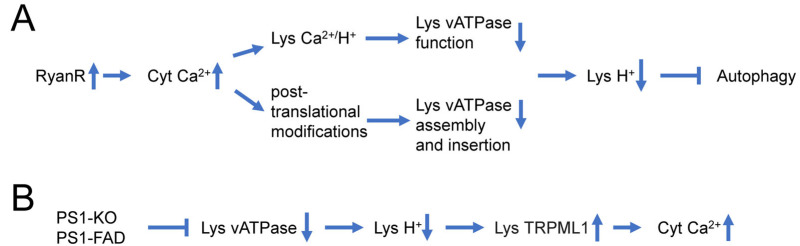
Causal relationship between dysregulation of Ca^2+^ signaling and impaired autophagy in AD neurons. (**A**) Supranormal RyanR-mediated Ca^2+^ release in AD neurons may decrease lysosomal vATPase expression levels through post-translational modifications that affect its assembly and insertion to the lysosome or disrupts vATPase function through stimulation of lysosomal Ca^2+^/H^+^ exchangers. Based on [24,99]. (**B**) Presenilin 1 (PS1) knockout or FAD linked mutations in PS1 lead to impaired glycosylation and instability of vATPase V0a1 subunit, further induce deficient lysosomal vATPase assembly and impaired its function and elevated lysosomal pH. Increased lysosomal pH induces abnormal Ca^2+^ efflux from lysosomes to cytoplasm by enhancing activity of lysosomal TRPML1 channels. Based on [28,29,91].

Recent reports suggest that dysregulation of Ca^2+^ signaling and impaired autophagy in AD neurons may have a causal relationship to each other. It has been suggested that lysosomal Ca^2+^ contributes to autophagy and is important for lysosomal degradation. Thus, intracellular Ca^2+^ distribution may affect lysosomal acidification (Figure 1A) [24]. Indeed, it has been reported that InsP_3_R preferentially associate with ER-lysosome contact sites and selectively deliver Ca^2+^ to lysosomes [98]. Ca^2+^ uptake by lysosomes may regulate lysosomal pH due to the activation of a lysosomal Ca^2+^/H^+^ exchanger (Figure 1A). For example, in hepatic cell HepG2, Ca^2+^ release from the ER caused a disruption of the lysosomal acidity and impaired protein degradation [99]. Recently increased Ca^2+^ release via RyanRs was reported to be associated with reduced expression of the lysosome proton pump vacuolar-ATPase (vATPase) subunits (V1B2 and V0a1), which cause lysosome deacidification and disrupt proteolytic activity. These findings are reported in both AD mouse models and human-induced neurons (HiN) [24] (Figure 1A). Normalizing AD-associated aberrant RyanR Ca^2+^ signaling with the negative allosteric modulator, dantrolene (Ryanodex), restored vATPase levels, lysosomal acidification and proteolytic activity, and autophagic clearance of intracellular protein aggregates in AD neurons [24]. These results directly implicate intracellular Ca^2+^ dysregulation in altering lysosomal vATPase expression levels and reinforces the important role of inter-organelle Ca^2+^ communication for vATPase trafficking to lysosomes, assembly of both domains, and lysosomal acidification (Figure 1A).

In addition to the effects of intracellular Ca^2+^ signaling on lysosomal acidification, there are also reports that suggest that changes in lysosomal acidification may also affect intracellular Ca^2+^ signaling (Figure 1B) [29]. Defects in V-ATPase targeting to lysosomes and lysosomal acidification were reported for PS1 knockout and FAD mutant cells [91]. It has been further reasoned that in addition to ER, lysosomes may also act as Ca^2+^ signaling organelles and that TRPML channels may mediate Ca^2+^ release from lysosomal compartments. It has been reported that lysosomal Ca^2+^ efflux through TRPML1 can trigger membrane fusion/fission events and regulate membrane trafficking [100], and that impairment in the TRPML1 function leads to various lysosomal storage diseases [101]. Moreover, it has been reported that impaired lysosomal acidification in presenilin knockout neurons causes an overactivation of lysosomal TRPMl1 channels [28,29], suggesting that impaired lysosomal acidification may contribute to dysregulated Ca^2+^ signaling in PS1 knockout and FAD mutant neurons (Figure 1B).

**Figure 2:**
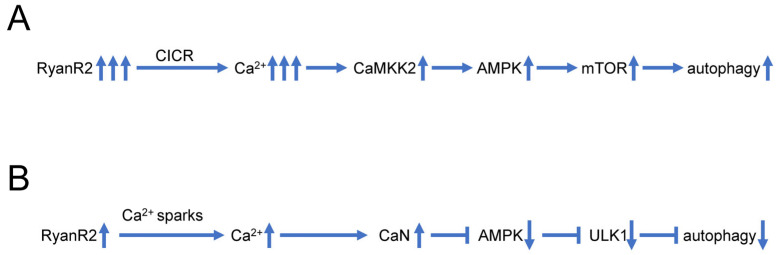
Biphasic effects of RyanR2-mediated Ca^2+^ signals on neuronal autophagy. (**A**) Evoked or stimulated Ca^2+^ release via RyanR2 leads to significant elevation of cytosolic Ca^2+^ levels, resulting in activation of CAMKK2-AMPK-mTOR pathway that promotes autophagy. Based on [102,103]. (**B**) Ca^2+^ sparks resulting from spontaneous basal activity of RyanR2 lead to modest elevation of cytosolic Ca^2+^ levels, sufficient to stimulate CaN and inhibit AMPK/ULK1 pathway, resulting in inhibition of autophagy. Based on [25,27].

Some recent findings also suggest that dysregulated Ca^2+^ signaling may play a more direct role in control of neuronal autophagy, independently from impaired lysosomal acidification (Figure 2A). In experiments with hippocampal neural stem (HCN) cells, it was shown that RyanR agonist caffeine significantly promoted the autophagic death of insulin-deficient HCN cells, and treatment with the RyanR inhibitor dantrolene prevented the induction of autophagy following insulin withdrawal [102]. Moreover, CRISPR/Cas9-mediated knockout of the RyanR3 gene in HCN cells abolished autophagic cell death [102]. Neferine, a natural alkaloid from Nelumbo nucifera, was reported to induce autophagy through Ulk-1-PERK and AMPK-mTOR signaling pathways in cancer cells which involved RyanRs activation [103]. Similarly, high doses of propofol (a commonly used intravenous anesthetic) can induce cytotoxicity in cortical progenitor cells, and data showed that blocking both InsP3R and RyanR can reduce autophagy and increase cell viability, suggesting that RyanR mediated excessive autophagy plays an important role in propofol induced toxicity [104]. All these studies suggest that excessive Ca^2+^ release via RyanR stimulates autophagy, most likely by stimulating the CaMKK2-AMPK-mTOR signaling pathway [102,103] (Figure 2A).

However, some reports also suggest that basal RyanR activity may actually inhibit autophagic flux (Figure 2B). Indeed, pharmacological inhibition of RyanR augmented autophagic flux in ectopic RyanR-expressing models such as HEK293 cells transfected with RyanR constructs or C2C12 myoblasts [25]. These studies have been performed using pharmacological modulators of RyanR activity that may exert off-target effects. In our recent studies, we took advantage of RyanR2-E4872Q knock-in mouse models (EQ) to test the importance of RyanR2 in control of neuronal autophagy in wild type and AD neurons. In EQ mice, the basal RyanR2-mediated Ca^2+^ influx is reduced due to shortened open channel time [105]. In our studies, we discovered augmented autophagic flux in primary hippocampal neuron cultures in a RyanR2-E4872Q knock-in mouse model (EQ) [27] (Figure 2B). Strikingly, we discovered thar EQ mutation is able to repair autophagic defects in two different AD mouse models (APPKI and APPPS1) [27]. Based on a series of additional pharmacological experiments, we demonstrated that overactivation of RyanR2 in AD neurons leads to persistent overactivation of cytosolic calcineurin (CaN), which is able to suppress autophagy by inhibiting AMPK/ULK1 pathways [27] (Figure 2B). Indeed, we have been able to achieve similar restoration of autophagic flux in AD neurons by inhibiting RyanR or by directly inhibiting CaN [27].

To reconcile these results, we propose that low levels of basal Ca^2+^ determined by spontaneous activity of RyanR2 primarily stimulates CaN-AMPK-ULK1 pathways to inhibit autophagy as we discovered [27] (Figure 2B), but high levels of Ca^2+^ during evoked or stimulated Ca^2+^ release led to the activation of the CAMKK2-AMPK-mTOR pathway and promoted autophagy as has been reported [102,103] (Figure 2A). Both CaN and CaMKK2 are regulated by Ca^2+^/calmodulin, and differential modulation of CaN and CaMKII by low and high Ca^2+^ elevations has already been described in the context of synaptic plasticity [106,107]. The model that we propose (Figure 2) has important implications for AD pathogenesis. Reduced autophagy impairs clearance of APP and APP proteolytic fragments, eventually leading to the accumulation of soluble Aβ42 oligomers, amyloid plaques and impaired synaptic plasticity. Indeed, in our studies, we demonstrated that the genetic cross of EQ mice with AD mouse models (APPKI and APPPS1) was able to reduce levels of accumulated Aβ, reduce amyloid plaque loads and repair LTP defects in these mice [27]. These results suggest that the beneficial effects of RyanR and CaN inhibitors in AD may at least in part be explained by their ability to repair autophagic defects.

**Figure 3:**
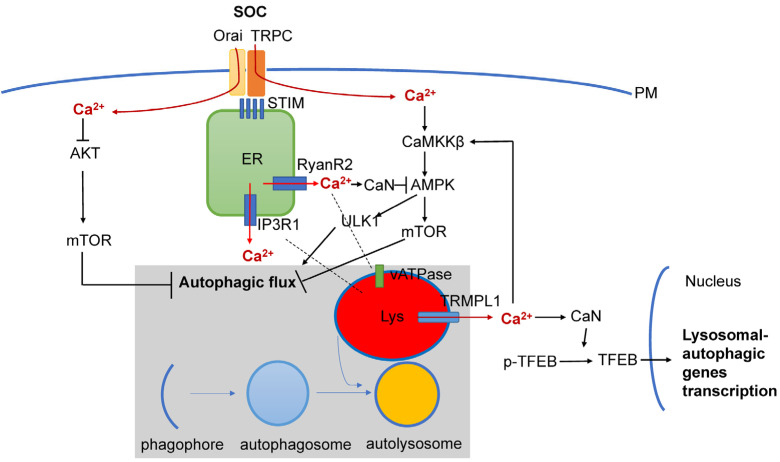
Interplay between Ca^2+^ signaling and neuronal autophagy in AD. Store-operated Ca^2+^ influx (SOC), Ca^2+^ release from ER via InsP_3_R1 and RyanR2 channels and Ca^2+^ release form lysosomal compartment via TRPM1 channel set basal levels of cytosolic Ca^2+^. Cytosolic Ca^2+^ controls autophagic pathways by directly acting on AKT, by modulating activity of CaMKKβ or CaN/AMPK/ULK1 and by modulating expression levels of genes involved in lysosomal and autophagic function via TFEB transcription factor. Dysregulation of Ca^2+^ signaling in AD neurons is closely linked with dysregulation of lysosomal function and autophagic pathways.

In addition to RyanR2-mediated pathways (Figure 2), there are also other Ca^2+^ signaling pathways that are connected with autophagy that may also play important roles in AD pathogenesis (Figure 3). It was shown that Ca^2+^ release through lysosomal TRMPL1 channels can activate CaN, leading to the dephosphorylation of TFEB transcription factors and resulting in increased expression of lysosomal and autophagic genes [108]. TRPML1 activity can also modulate autophagy through the activation of the CaMKKβ/AMPK/ULK1/VPS34 pathway [109]. Recently, functional defects in the TRPML1 Ca^2+^ channels were identified in LOAD patients’ brain samples [110]. In the same study it was shown that decreased TRPML1-mediated lysosomal Ca^2+^ released in a neuronal apoE4 iPSC model can be reduced by treatment with ML-SA1, a small-molecule TRPML1 agonist [110]. It was also shown that TFEB mediates mutant tau releases in iPSC-derived neurons in a TRPML1-dependent manner [111]. All of these results suggest that TRPML1 may also be considered as a potential therapeutic treatment for AD (Figure 3).

Store-operated Ca^2+^ entry (SOCE) is a major calcium-entry pathway in non-excitable cells, but it is also an important Ca signaling pathway in neurons and plays an important role in AD pathogenesis [112,113] (Figure 3). ER Ca^2+^ depletion induces ER calcium sensors STIM1 or STIM2 to translocate to the plasma membrane, where they activate Orai and/or TRPC channels, causing Ca^2+^ entry. In non-neuronal cell types, STIM1/ORAI1/TRPC have been shown to regulate autophagy and apoptosis [17], and recently, STIM1 mediated SOCE was reported to induce autophagy through AKT/mTOR pathways in hippocampal neurons under hypoxic conditions [114]. Dexmedetomidine (DEX) was reported to exert neuroprotective effects in PC12 cells through STIM1/Orai1 signaling pathways by regulating autophagy and apoptosis [115]. Previously we reported that STIM2/ORAI2/TRPC6 was impaired in AD [116], and TRPC6 was reported to induce autophagy through CaMKKβ-AMPK-mTOR pathways [117]. Thus, it will be interesting to explore the role of STIM2/ORAI2/TRPC6 impaired function in AD autophagy dysfunction. Moreover, it has been reported that ER stress can degrade STIM2 through autophagy, represses SOCE and disrupts dendrite arbor in primary neuronal cultures [118]. In the same paper it was also proposed that autophagy represses SOCE by degrading STIM proteins, leading to synapse loss in AD [119], providing additional potential feedback between Ca^2+^ signaling and autophagy in AD neurons.

## 6. Conclusions

Dysregulation of intracellular neuronal Ca^2+^ signaling and impairment of neuronal autophagy are two well-established phenomenon observed in AD neurons. Until recently studies of these two signaling pathways proceeded largely independently of each other. However, several recent papers point to a causal connection between them. Some results suggest that defects in lysosomal acidification may contribute to Ca^2+^ signaling defects by causing an overactivation of lysosomal TRPML1 channels [29]. Some data suggest that enhanced activity of RyanR may lead to defects in lysosomal acidification by affecting the activity of lysosomal V-ATPase [24]. Our recent findings suggest that basal Ca^2+^ released by RyanR2 may control steady-state levels of autophagy via CaN-AMPK-ULK1 pathways and that overactivation of RyanR2 in AD may lead to the overstimulation of CaN and the inhibition of autophagic flux [27]. Further studies will be needed to dissect relationships between intracellular Ca^2+^ dysregulation and autophagy in AD, but obtained results already offer some novel mechanistic insight and may potentially lead to the identification of novel therapeutic methods for treating AD and potentially other neurodegenerative disorders.

## Data Availability

Not applicable.
